# Development and validation of a machine learning-based model for identifying liver fibrosis in individuals with prior *Schistosoma japonicum* infection: a step toward precision management

**DOI:** 10.1186/s40249-026-01485-y

**Published:** 2026-08-20

**Authors:** Tao Wang, Yi Jiang, JianFeng Zhang, WeiMin Li, HuiXin Xue, HaiYong Hua, Wei Wang, Kun Yang

**Affiliations:** 1 Key Laboratory of National Health Commission of Parasitic Disease Prevention and Control, Jiangsu Provincial Key Laboratory of Parasites and Vector Control Technology, Jiangsu Institute of Parasitic Diseases, Wuxi, 214064 China; 2 School of Public Health, Nanjing Medical University, Nanjing, 211166 China; 3 Department of Ultrasonography, Affiliated Hospital of Jiangnan University, Wuxi, 214064 China

**Keywords:** *Schistosoma japonicum*, Liver fibrosis, Machine learning, Non-invasive diagnosis

## Abstract

**Background:**

Liver fibrosis caused by *Schistosoma japonicum* may continue to progress even following successful praziquantel chemotherapy. Although liver biopsy remains the gold standard for diagnosis of liver fibrosis, its invasiveness limits clinical applications. Conventional non-invasive indices, such as aspartate aminotransferase to platelet ratio index (APRI) and fibrosis-4 (FIB-4) index, often show suboptimal performance in schistosomiasis-related cases. This study aimed to develop and validate a machine learning (ML)-based model using epidemiological and laboratory data to identify liver fibrosis in individuals with prior *S. japonicum* infection, as a step toward precision management.

**Methods:**

Data were obtained from the Jiangsu *S. japonicum* Infection Cohort (Jiangsu Province, China), involving 6158 participants with a documented history of infection who completed follow-up assessments during 2021–2022. Modeling variables were selected using LASSO regression and variance inflation factor analysis. Five ML models (k-nearest neighbor, logistic regression, support vector machine, decision tree, and extreme gradient boosting (XGBoost)) were evaluated. Model performance was compared against APRI and FIB-4 using the area under the receiver operating characteristic curve (AUC), decision curve analysis (DCA), and shapley additive explanations (SHAP) for interpretability.

**Results:**

In the validation set, the XGBoost model demonstrated the highest diagnostic performance with an AUC of 0.854 (95% confidence interval (*CI):* 0.826–0.881), outperforming other ML models (AUC range: 0.609–0.776). This was significantly superior to FIB-4 (AUC = 0.514) and APRI (AUC = 0.516) (*P* < 0.001). DCA confirmed that the XGBoost model provided a substantial clinical net benefit across a broad range of threshold probabilities. According to Shapley additive explanations analysis, variables such as alcohol intake (mean SHAP value = 0.401), gamma-glutamyl transferase (0.295), and triglycerides (0.292) were the most influential predictors of liver fibrosis risk.

**Conclusions:**

The XGBoost-based model offers a robust, non-invasive tool for identifying liver fibrosis in individuals with prior *S. japonicum* infection, with alcohol intake, gamma-glutamyl transferase, and triglycerides identified as the critical predictors. By outperforming traditional indices and leveraging routinely available data, this model represents a promising advancement toward precision management of individuals with prior *S. japonicum* infection, offering a scalable approach for early intervention, risk‑stratified assessment, and monitoring of hepatic morbidity in post‑transmission settings.

## Background

Schistosomiasis ranks second only to malaria as the most devastating parasitic disease across the world in terms of its prevalence, public health impact and socioeconomic burden [1]. Currently, an estimated 253.7 million people worldwide required preventive treatment for schistosomiasis in 2024, contributing to approximately 14,353 deaths annually [2]. There are three major species of the genus *Schistosoma* infecting humans: *S. japonicum* and *S. mansoni*, causing intestinal schistosomiasis, and *S. haematobium,* causing urogenital schistosomiasis [3]. *S. japonicum* infection is proven to result in more severe health problems than infection with other species [4]. Deposition of Schistosoma japonicum eggs in the liver triggers granulomatous inflammation, which can progress to hepatic fibrosis, hepatomegaly, portal hypertension, and hypersplenism, and can even be fatal in severe cases [5].

China was once among the countries most severely affected by schistosomiasis japonica, with a cumulative total exceeding 10 million reported cases nationwide [6,7]. Following decades of integrated control efforts, transmission levels have been reduced to a historic low, and nationwide interruption of schistosomiasis transmission was achieved in 2023 (defined as five consecutive years with zero documented infections among humans, livestock, and snail intermediate hosts). The country is now moving toward the goal of disease elimination [8]. Currently, most schistosomiasis patients are identified without active infection [9]. However, accumulating evidence suggests that praziquantel treatment does not consistently halt the progression of hepatic fibrosis in all infected individuals. Even following the successful eradication of adult worms and the neutralization of viable eggs, persistent fibrogenesis can occur in certain hosts, leading to severe clinical sequelae [5, 10–12]. Follow-up studies in transmission-interrupted areas have demonstrated that patients with previous infections can still develop advanced schistosomiasis. Even without the risk of reinfection after treatment, their condition can continue to worsen [10–12]. A history of *S. japonicum* infection has been shown to correlate more strongly with the severity of liver parenchymal fibrosis [odds ratio (*OR*)= 1.84, 95% confidence interval (*CI*): 1.30–2.60] than current active infection (*OR* = 1.40, 95% *CI*: 1.03–1.90) [10]*.* Notably, approximately 30% (86/292) of individuals with prior infection eventually develop severe liver fibrosis [13].

Given that early-stage liver fibrosis is potentially reversible through timely anti-fibrotic interventions [14], the development of simple, reliable, and non-invasive diagnostic tools is central to improving prognosis and alleviating the public health burden due to schistosomiasis-related liver fibrosis [15]. Although liver biopsy remains the clinical gold standard for diagnosing and staging fibrosis, its application is greatly constrained by its invasive nature, potential for severe complications (e.g., hemorrhage and sepsis), and low patient compliance [16]. Consequently, non-invasive modalities, particularly medical imaging and serological biomarkers, have received increasing attention [17]. Nevertheless, single hematological markers are challenging because of their low sensitivity and specificity for precise diagnosis [18]. Multivariable indices have been therefore proposed, notably for viral hepatitis cohorts, such as aspartate aminotransferase (AST) to platelet (PLT) ratio index (APRI) [19] and fibrosis-4 (FIB-4) index [20]; however, their diagnostic performance remains inconsistent and frequently suboptimal among schistosomiasis patients [21,22]. Ultrasonography remains a common tool for diagnosing liver fibrosis, but its clinical utility is limited by high inter-observer variability due to complex criteria, equipment differences, and operator subjectivity [23,24]. These limitations reduce the effectiveness of the aforementioned tools for diagnosis of subtle, early-stage fibrosis.

Machine learning (ML) offers a transformative approach to medical diagnostics by decoding complex, non-linear relationships within high-dimensional clinical data [25,26]. ML-based algorithms utilizing routine epidemiological and laboratory variables have demonstrated superior accuracy for fibrosis staging among patients with chronic hepatitis B and metabolic dysfunction-associated steatotic liver disease [27–31]. However, there is little knowledge on the use of ML in schistosomiasis-related liver fibrosis [32,33]. This is particularly true for individuals with a history of *S. japonicum* infection, whose clinical profiles exhibit significant heterogeneity and require the integration of a broader spectrum of predictors.

The present study was designed to develop and validate an ML-based diagnostic model for liver fibrosis in a cohort of individuals with prior *S. japonicum* infection, combining both epidemiological and routine laboratory indicators. By comparatively evaluating multiple ML models against conventional indices, we seek to establish a robust, non-invasive screening tool. Such a model may facilitate accurate clinical assessment, enable earlier interventions, and support the long-term monitoring of hepatic morbidity among those with prior *S. japonicum* infection.

## Methods

### Study population

The current study adopted a cross-sectional design using data collected from the 2021–2022 follow-up study of the Jiangsu *S. japonicum* Infection Cohort., a prospective longitudinal cohort based in Jiangsu Province, China. The cohort was established in 2017 to investigate long-term health trajectories among individuals with prior S. japonicum infection in this historically hyperendemic region for schistosomiasis [34,35]. Inclusion criteria for the current analysis were: (1) a documented history of *S. japonicum* infection confirmed by local parasitological records; (2) completion of standardized anti-schistosomiasis praziquantel therapy; and (3) willingness to undergo comprehensive physical and ultrasonographic examinations. To eliminate potential hepatic confounders, the following exclusion criteria were strictly applied: (1) presence of severe autoimmune diseases; (2) co-infection with hepatitis B virus, hepatitis C virus or other viral hepatitis viruses; (3) diagnosis of alcohol-associated or metabolic dysfunction-associated steatotic liver disease; and (4) history of primary hepatic malignancy.

### Data collection and variable definitions

All clinical assessments, laboratory tests, and ultrasonographic examinations were performed simultaneously during the 2021–2022 follow-up visit. Participants underwent standardized assessments by well-trained healthcare professionals. Demographic and lifestyle data, including age, gender, and alcohol consumption habits, were obtained via structured face-to-face interviews. Anthropometric measurements included body weight, height, waist circumference, and resting blood pressure [systolic blood pressure (SBP); diastolic blood pressure (DBP)] were recorded. Body mass index (BMI) was calculated as weight in kilograms divided by height in meters squared (kg/m^2^). Laboratory parameters were quantified from fasting venous blood samples. Routine blood tests included red blood cell (RBC), white blood cell (WBC), hemoglobin, PLT, fasting plasma glucose, and lipid profiles [total cholesterol, triglyceride, low-density lipoprotein cholesterol (LDL-C), and high-density lipoprotein cholesterol (HDL-C)]. Liver function markers included alanine aminotransferase (ALT), AST, gamma-glutamyl transferase (GGT), total protein, albumin, globulin, and the albumin/globulin (A/G) ratio. Hyaluronic acid was measured as a marker of hepatic fibrosis. Additionally, two non-invasive fibrosis scores were calculated using the following formulas:

$\mathrm{A}\mathrm{P}\mathrm{R}\mathrm{I}=\frac{\frac{\mathrm{A}\mathrm{S}\mathrm{T}}{{\mathrm{ULN}}_{\mathrm{A}\mathrm{S}\mathrm{T}}}\times 100}{\mathrm{P}\mathrm{L}\mathrm{T}}$, FIB-4 = $\frac{\mathrm{A}\mathrm{g}\mathrm{e}\times \mathrm{A}\mathrm{S}\mathrm{T}}{\mathrm{P}\mathrm{L}\mathrm{T}\times \sqrt{\mathrm{A}\mathrm{L}\mathrm{T}}}$, where ULN denotes the upper limit of normal for AST.

### Ultrasonographic assessment

The primary outcome was presence of liver fibrosis, assessed via high-resolution abdominal ultrasonography. Examinations were performed by two senior radiologists blinded to participants' clinical data. Liver parenchymal changes were graded according to the World Health Organization diagnostic criteria for *S. japonicum*-related morbidity [36]. The fibrosis status was classified into four grades (0 to III). For classification, the outcome was dichotomized into a non-fibrosis group (Grade 0) and a liver fibrosis group (Grades I, II, and III). Any discordance between the two radiologists was resolved by a third hepatologist to ensure diagnostic accuracy.

### Statistical analysis

**Fig. 1:**
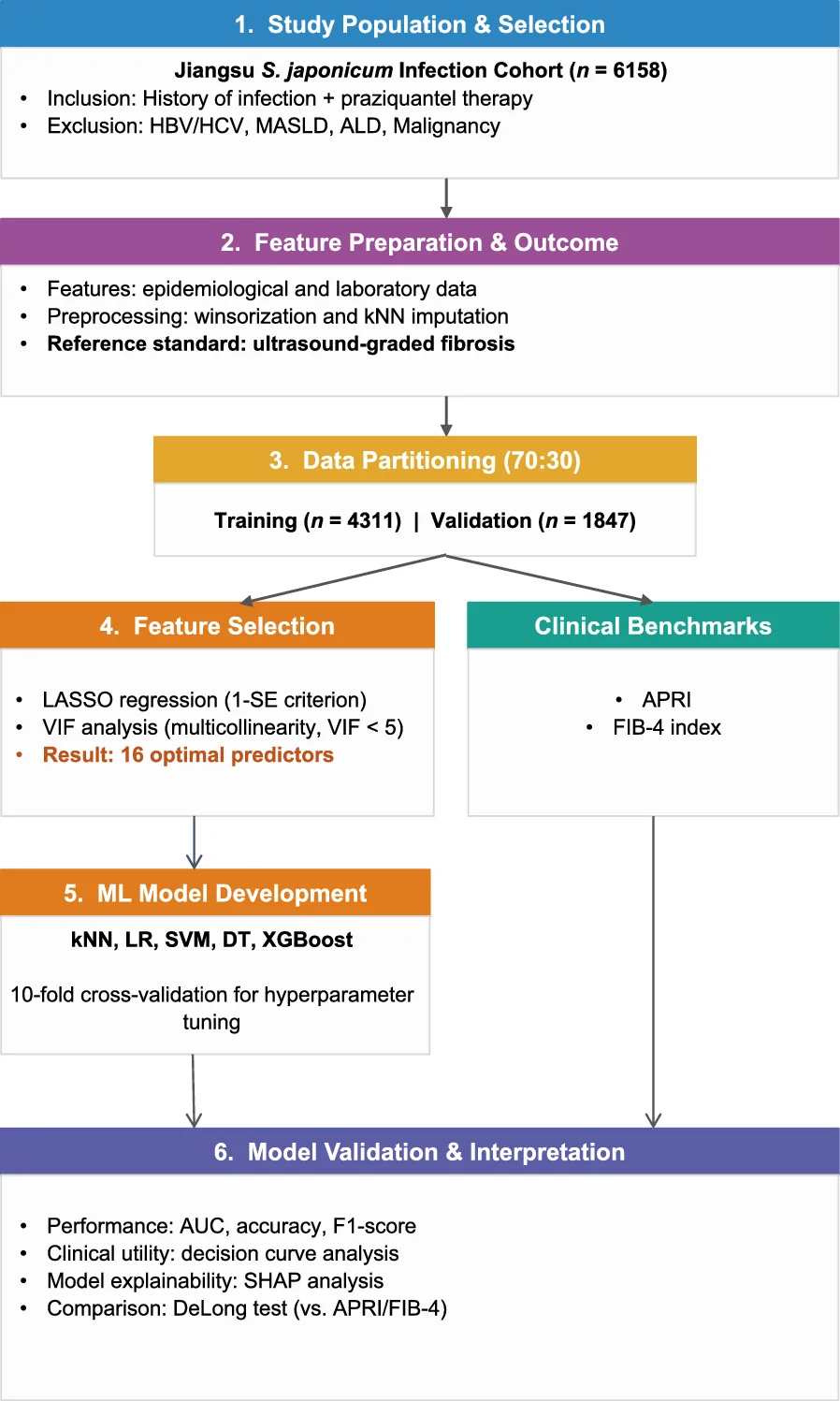
Study pipeline for model development and validation. HBV: hepatitis B virus; HCV: hepatitis C virus; ALD: alcohol-associated liver diseases; MASLD: metabolic dysfunction-associated steatotic liver disease; APRI: aspartate aminotransferase to platelet ratio index; FIB-4: fibrosis-4 index; VIF: variance inflation factor; kNN*:* k-nearest neighbor, LR: logistic regression, SVM: support vector machine, DT: decision tree; XGBoost: extreme gradient boosting; AUC: area under the receiver operating characteristic curve; SHAP: shapley additive explanations

Continuous variables were expressed as mean ± standard deviation (*SD*) or median (interquartile range, IQR), while categorical variables were presented as frequencies (*n*, %). Inter-group comparisons were conducted using Student's *t*-test, Mann–Whitney *U* test, or chi-square test, as appropriate. Data preprocessing involved three key steps: (1) Winsorization was applied to mitigate the influence of extreme outliers; (2) Missing values were imputed using the k-nearest neighbors (kNN) algorithm to maximize data utilization; (3) The dataset was partitioned into a training set (70%) and a validation set (30%) using stratified random sampling based on the primary outcome. For reproducibility, the partitioning was performed with a fixed random seed within the R caret package. To ensure a rigorous and unbiased evaluation, the entire model development process was confined to the training set using tenfold cross-validation. The validation set was strictly held out and utilized only for the final assessment of model performance and generalization capability. Variable selection was performed using LASSO regression with tenfold cross-validation (selecting the lambda within 1 standard error), followed by variance inflation factor (VIF) analysis to exclude variables with multicollinearity. A conservative threshold of VIF > 5 (rather than the conventional cutoff of 10) was applied to further minimize multicollinearity among predictors. Given the diversity of ML approaches, we evaluated five representative models: k-nearest neighbor, logistic regression (LR), support vector machine (SVM), decision tree (DT), and extreme gradient boosting (XGBoost). These models encompass linear, non-linear, and ensemble learning architectures. Following established practices in recent liver fibrosis research [37–40], this multi-model strategy was adopted to ensure a comprehensive comparison and to identify the optimal architecture for our dataset. To enhance model robustness and prevent overfitting, we performed hyperparameter optimization using a grid search strategy coupled with tenfold cross-validation on the training set. To address the inherent class imbalance in liver fibrosis stages, synthetic minority over-sampling technique was integrated within the cross-validation loop to balance the target classes. The final model, configured with the optimal parameters, was then evaluated on an independent validation set to assess its generalizability. The diagnostic performance of models was evaluated in the validation set using the area under the receiver operating characteristic (ROC) curve (AUC), accuracy, sensitivity, specificity, positive predictive value (PPV), negative predictive value (NPV), and F1-score. DeLong test was used to compare ROC curves between the optimal model and conventional indices (APRI/FIB-4). Clinical utility was assessed with decision curve analysis (DCA). Finally, model interpretability was achieved using shapley additive explanations (SHAP) analysis to identify key risk factors. A two-sided *P*-value of < 0.05 was considered statistically significant. All statistical analyses and data processing were performed using R package version 4.4.1 (The R Foundation for Statistical Computing; Vienna, Austria). The overall study pipeline is illustrated in Fig. 1.

### Ethical statement

The study was approved by the Ethics Review Committee of Jiangsu Institute of Parasitic Diseases (approval number: JIPD-(2023) 34). All procedures were conducted in accordance with the Declaration of Helsinki, and international and national laws, regulations and guideline for human studies. Written informed consent was obtained from all participants, following a detailed description of the purpose of and potential benefits from the study. Clinical trial number: not applicable.

## Results

### Baseline subject characteristics

A total of 6158 individuals with prior *S. japonicum* infection were enrolled in this study. The cohort had a median age of 64 (IQR: 59–68) years, and included 3518 (57.1%) males. Liver fibrosis was identified in 4314 individuals (70.1%). The study population was randomly partitioned into a training set and a validation set in a 7:3 ratio. The baseline demographic and clinical profiles were well-balanced between the two groups (all *P* > 0.05) (Table 1), indicating that the data splitting was mostly randomized. Additionally, all the data had comparable distributions from the subsequent modeling.

**Table 1:** Baseline demographic and clinical characteristics of subjects in the training and validation sets

Variables	Overall (*n* = 6158)	Training set (*n* = 4311)	Validation set (*n* = 1847)	P^*^
Age (years)	64.00 (59.00, 68.00)	64.00 (59.00, 68.00)	64.00 (59.00, 68.00)	0.503
Sex (Male) (%)	3518 (57.1)	2471 (57.3)	1047 (56.7)	0.666
Height (cm)	161.20 (155.50, 167.00)	161.20 (155.70, 167.00)	161.30 (155.00, 167.00)	0.553
Weight (kg)	63.10 (56.50, 70.00)	63.10 (56.50, 70.00)	63.00 (56.20, 70.00)	0.288
BMI (kg/m^2^)	24.44 ± 3.15	24.46 ± 3.13	24.41 ± 3.19	0.572
Waist circumference (cm)	83.00 (78.00, 89.00)	83.10 (78.00, 89.00)	83.00 (78.00, 89.00)	0.855
SBP (mmHg)	138.00 (126.00, 150.00)	138.00 (126.00, 150.00)	138.00 (126.00, 151.00)	0.316
DBP (mmHg)	82.00 (76.00, 90.00)	82.00 (76.00, 90.00)	82.00 (77.00, 90.00)	0.984
RBC (10^12^/L)	4.54 (4.23, 4.85)	4.54 (4.24, 4.84)	4.53 (4.21, 4.86)	0.771
Hemoglobin (g/L)	140.00 (130.00, 150.00)	140.00 (130.00, 150.00)	140.00 (130.00, 151.00)	0.944
WBC (10⁹/L)	5.56 (4.68, 6.61)	5.57 (4.68, 6.64)	5.52 (4.69, 6.58)	0.308
PLT (10⁹/L)	175.00 (140.00, 213.00)	174.00 (139.00, 213.00)	176.00 (142.00, 212.00)	0.596
Glucose (mmol/L)	5.50 (5.05, 6.10)	5.49 (5.03, 6.10)	5.52 (5.07, 6.11)	0.345
Total cholesterol (mmol/L)	4.81 (4.21, 5.46)	4.82 (4.20, 5.47)	4.80 (4.22, 5.45)	0.891
Triglyceride (mmol/L)	1.25 (0.88, 1.88)	1.26 (0.88, 1.88)	1.24 (0.89, 1.85)	0.73
LDL-C (mmol/L)	2.68 (2.23, 3.23)	2.68 (2.23, 3.22)	2.68 (2.22, 3.24)	0.762
HDL-C (mmol/L)	1.44 (1.23, 1.70)	1.44 (1.23, 1.71)	1.45 (1.23, 1.70)	0.406
ALT (U/L)	20.84 (16.00, 27.78)	21.00 (16.00, 28.00)	20.30 (16.00, 27.00)	0.089
AST (U/L)	23.00 (19.30, 28.00)	23.00 (19.20, 28.00)	23.00 (19.40, 27.70)	0.973
GGT (U/L)	24.40 (17.30, 39.00)	24.58 (17.50, 39.00)	24.00 (17.00, 39.00)	0.191
Total protein (g/L)	74.30 (71.10, 77.50)	74.30 (71.02, 77.40)	74.40 (71.30, 77.60)	0.384
Albumin (g/L)	46.80 (44.80, 48.70)	46.80 (44.80, 48.70)	46.80 (44.83, 48.78)	0.551
Globulin (g/L)	27.50 (24.80, 30.47)	27.40 (24.80, 30.40)	27.60 (24.80, 30.70)	0.425
A/G ratio	1.70 (1.50, 1.90)	1.70 (1.51, 1.90)	1.70 (1.50, 1.90)	0.242
Hyaluronic acid	73.91 (52.98, 101.45)	73.92 (53.06, 101.63)	73.87 (52.85, 100.78)	0.805
Alcohol intake (%)				0.585
Never	4097 (66.5)	2852 (66.2)	1245 (67.4)	
Current	1308 (21.2)	930 (21.6)	378 (20.5)	
Occasional	753 (12.2)	529 (12.3)	224 (12.1)	
Fibrosis (%)				1
Non-fibrosis	1844 (29.9)	1291 (29.9)	553 (29.9)	
Liver fibrosis	4314 (70.1)	3020 (70.1)	1294 (70.1)	

BMI: body mass index; SBP: systolic blood pressure; DBP: diastolic blood pressure; RBC: red blood cell; WBC: white blood cell; PLT: platelet; LDL-C: low-density lipoprotein cholesterol; HDL-C: high-density lipoprotein cholesterol; ALT: alanine aminotransferase; AST: aspartate aminotransferase; GGT: gamma-glutamyl transferase; A/G ratio: albumin/globulin ratio;

Data are presented as mean ± SD, median (IQR), or n (%)

^*^Inter-group comparisons were conducted using Student's *t*-test, Mann–Whitney *U* test, or chi-square test, as appropriate

### Variable selection

**Fig. 2:**
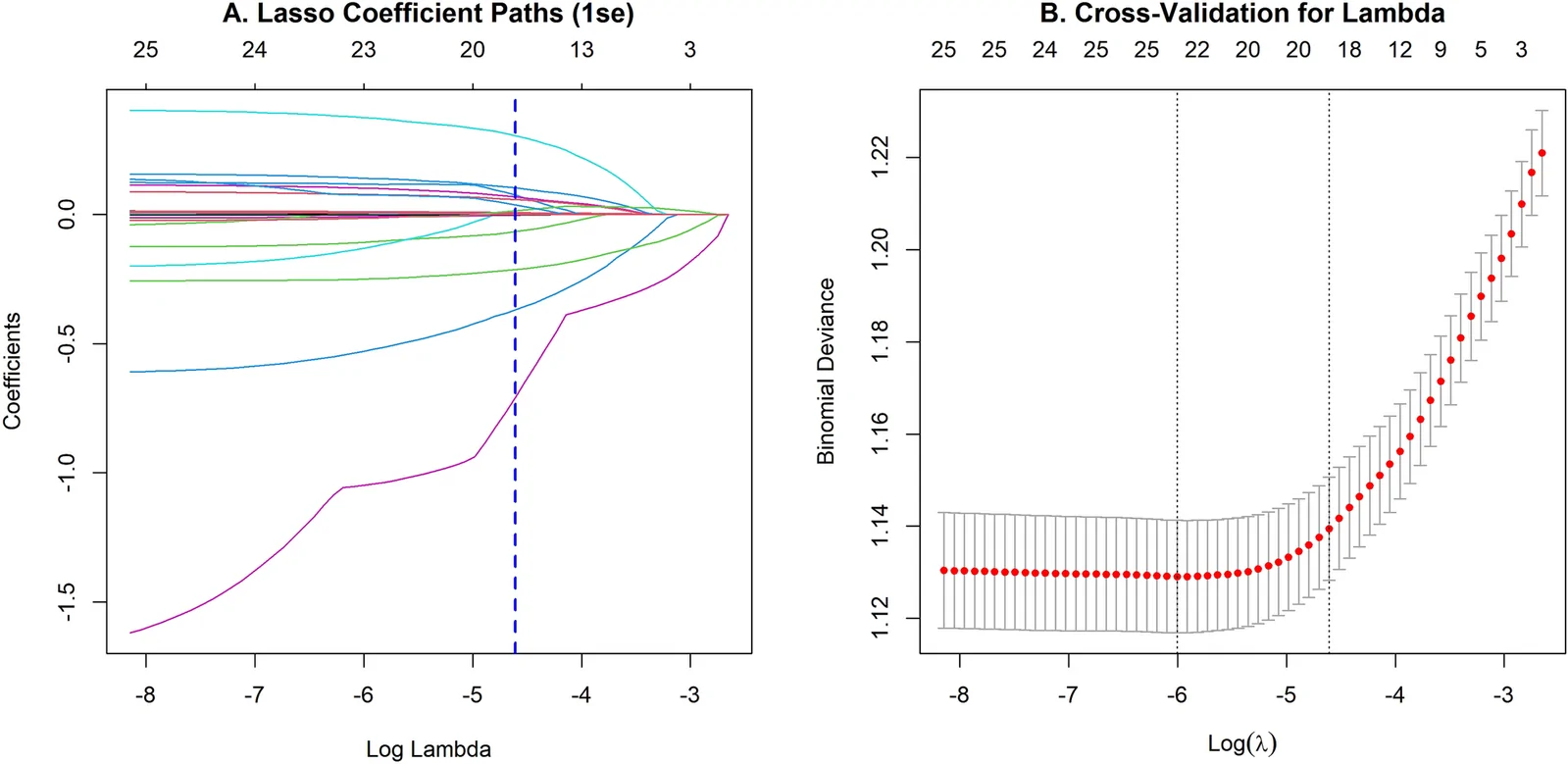
Variable selection using the LASSO logistic regression model. **A** LASSO coefficient profiles of the candidate predictors. Each colored line represents the coefficient of an individual variable. The vertical blue dashed line is drawn at the optimal value chosen by one standard error (1se) criterion; **B** Tuning parameter (lambda) selection in the LASSO model using tenfold cross-validation. The binomial deviance was plotted against log(lambda). The left vertical dotted line indicates the minimum binomial deviance (lambda-min), and the right vertical dotted line represents the one standard error of the minimum binomial deviance (lambda-1se)

Initially, 18 candidate predictors were identified using the LASSO binary LR model based on the one-standard error (1-SE) criterion (Fig. 2). Subsequently, multicollinearity diagnostics were performed to ensure the stability of the ML models. As summarized in Table 2, significant collinearity was detected among total protein, albumin, and the A/G ratio, with VIFs exceeding 5. Given the inherent mathematical dependency among these protein-related indices, we retained only the A/G ratio for the final multivariable model. Following this refinement, all remaining 16 variables demonstrated acceptable independence with VIFs below 5. The final multivariable model incorporated the following predictors: age, alcohol intake, weight, SBP, DBP, RBC, WBC, PLT, glucose, triglycerides, HDL-C, LDL-C, ALT, GGT, A/G ratio, and hyaluronic acid.

**Table 2:** Variance inflation factor analysis for variable selection

Variable	GVIF	*df*	GVIF^(1/(2*df))^	Adjusted_VIF
Age	1.20	1	1.10	1.20
Alcohol intake	1.25	2	1.06	1.12
Weight	1.32	1	1.15	1.32
SBP	1.70	1	1.30	1.70
DBP	1.73	1	1.32	1.73
RBC	1.28	1	1.13	1.28
WBC	1.22	1	1.10	1.22
PLT	1.19	1	1.09	1.19
Glucose	1.08	1	1.04	1.08
Triglycerides	1.24	1	1.11	1.24
LDL-C	1.09	1	1.04	1.09
HDL-C	1.20	1	1.10	1.20
ALT	1.26	1	1.12	1.26
GGT	1.38	1	1.17	1.38
Total protein	8.20	1	2.86	8.20
Albumin	8.74	1	2.96	8.74
A/G ratio	9.23	1	3.04	9.23
Hyaluronic acid	1.13	1	1.07	1.13

SBP: systolic blood pressure; DBP: diastolic blood pressure; RBC: red blood cell; WBC: white blood cell; PLT: platelet; LDL-C: low-density lipoprotein cholesterol; HDL-C: high-density lipoprotein cholesterol; ALT: alanine aminotransferase; GGT: gamma-glutamyl transferase; A/G ratio: albumin/globulin ratio; GVIF: generalized variance inflation factor; *df*: degrees of freedom

Adjusted VIF = (GVIF^(1/(2 × *df*))^)^2^. An adjusted VIF < 5 indicates the absence of significant multicollinearity

### Diagnostic performance of models

The XGBoost model demonstrated robust diagnostic performance, achieving the highest AUC of 0.928 (95% *CI*: 0.920–0.936) in the training set. Although a slight decrease in performance was observed in the validation set, the XGBoost model remained the top-performing predictor with an AUC of 0.854 (95% *CI*: 0.826–0.881). Detailed performance metrics for the validation set, including accuracy, sensitivity, specificity, PPV, NPV, and F1-score were 0.818 (95% *CI*: 0.772–0.854), 0.853 (95% *CI*: 0.772–0.923), 0.736 (95% *CI*: 0.662–0.814), 0.884 (95% *CI*: 0.856–0.913), 0.687 (95% *CI*: 0.583–0.797), and 0.868 (95% *CI*: 0.827–0.900), respectively (Table 3).

**Table 3:** Comparison of diagnostic performance among machine learning models and clinical indices in the training and validation sets

	AUC	Cutoff	Accuracy	Sensitivity	Specificity	PPV	NPV	F1 score
Training								
LR	0.674 (0.659–0.690)	0.469 (0.427–0.506)	0.668 (0.631–0.705)	0.724 (0.638–0.811)	0.538 (0.439–0.628)	0.786 (0.768–0.807)	0.459 (0.416–0.513)	0.752 (0.710–0.793)
SVM	0.806 (0.793–0.819)	0.553 (0.514–0.566)	0.745 (0.726–0.762)	0.760 (0.736–0.800)	0.709 (0.659–0.738)	0.859 (0.844–0.872)	0.560 (0.527–0.595)	0.807 (0.790–0.823)
kNN	0.836 (0.824–0.849)	0.478 (0.422–0.528)	0.733 (0.717–0.786)	0.701 (0.680–0.839)	0.807 (0.664–0.831)	0.895 (0.852–0.908)	0.538 (0.514–0.641)	0.785 (0.772–0.846)
DT	0.617 (0.603–0.634)	0.533 (0.533–0.533)	0.708 (0.696–0.721)	0.834 (0.821–0.848)	0.413 (0.389–0.439)	0.768 (0.755–0.780)	0.517 (0.485–0.544)	0.800 (0.791–0.809)
XGBoost	0.928 (0.920–0.936)	0.608 (0.571–0.647)	0.856 (0.837–0.873)	0.862 (0.821–0.904)	0.840 (0.788–0.877)	0.926 (0.907–0.942)	0.725 (0.675–0.787)	0.893 (0.876–0.909)
APRI	0.508 (0.494–0.528)	0.314 (0.187–0.529)	0.545 (0.378–0.675)	0.585 (0.179–0.922)	0.450 (0.103–0.855)	0.715 (0.698–0.742)	0.321 (0.301–0.351)	0.626 (0.288–0.798)
FIB-4	0.529 (0.510–0.546)	1.878 (1.208–2.506)	0.518 (0.422–0.658)	0.500 (0.247–0.855)	0.559 (0.188–0.811)	0.729 (0.706–0.758)	0.328 (0.304–0.373)	0.578 (0.370–0.778)
Validation								
LR	0.643 (0.605–0.683)	0.431 (0.317–0.568)	0.681 (0.572–0.751)	0.775 (0.503–0.931)	0.460 (0.297–0.726)	0.774 (0.736–0.831)	0.489 (0.374–0.647)	0.768 (0.619–0.842)
SVM	0.756 (0.723–0.788)	0.523 (0.435–0.630)	0.737 (0.671–0.783)	0.781 (0.647–0.892)	0.633 (0.501–0.780)	0.835 (0.796–0.879)	0.563 (0.450–0.690)	0.804 (0.737–0.850)
kNN	0.776 (0.741–0.801)	0.500 (0.472–0.528)	0.700 (0.672–0.736)	0.671 (0.632–0.715)	0.768 (0.713–0.820)	0.872 (0.844–0.900)	0.499 (0.454–0.548)	0.758 (0.731–0.791)
DT	0.609 (0.579–0.641)	0.533 (0.533–0.533)	0.710 (0.686–0.745)	0.858 (0.828–0.883)	0.364 (0.321–0.410)	0.760 (0.734–0.788)	0.521 (0.456–0.579)	0.806 (0.786–0.833)
XGBoost	0.854 (0.826–0.881)	0.585 (0.531–0.664)	0.818 (0.772–0.854)	0.853 (0.772–0.923)	0.736 (0.662–0.814)	0.884 (0.856–0.913)	0.687 (0.583–0.797)	0.868 (0.827–0.900)
APRI	0.516 (0.485–0.559)	0.328 (0.141–1.089)	0.572 (0.314–0.714)	0.646 (0.023–0.986)	0.397 (0.035–0.991)	0.727 (0.687–0.854)	0.359 (0.291–0.562)	0.638 (0.046–0.829)
FIB-4	0.514 (0.490–0.547)	1.852 (0.762–3.018)	0.538 (0.365–0.702)	0.553 (0.157–0.986)	0.501 (0.046–0.897)	0.731 (0.693–0.793)	0.350 (0.290–0.588)	0.590 (0.261–0.820)

kNN: k-nearest neighbor; LR: logistic regression; SVM: support vector machine; DT: decision tree; XGBoost: extreme gradient boosting; AUC: area under the receiver operating characteristic curve; PPV: positive predictive value; NPV: negative predictive value; APRI: aspartate aminotransferase-to-platelet ratio index; FIB-4: fibrosis-4 index

Values are presented as Mean with 95% confidence interval based on 100 bootstrap iterations. The optimal cutoff was determined using the Youden index

**Fig. 3:**
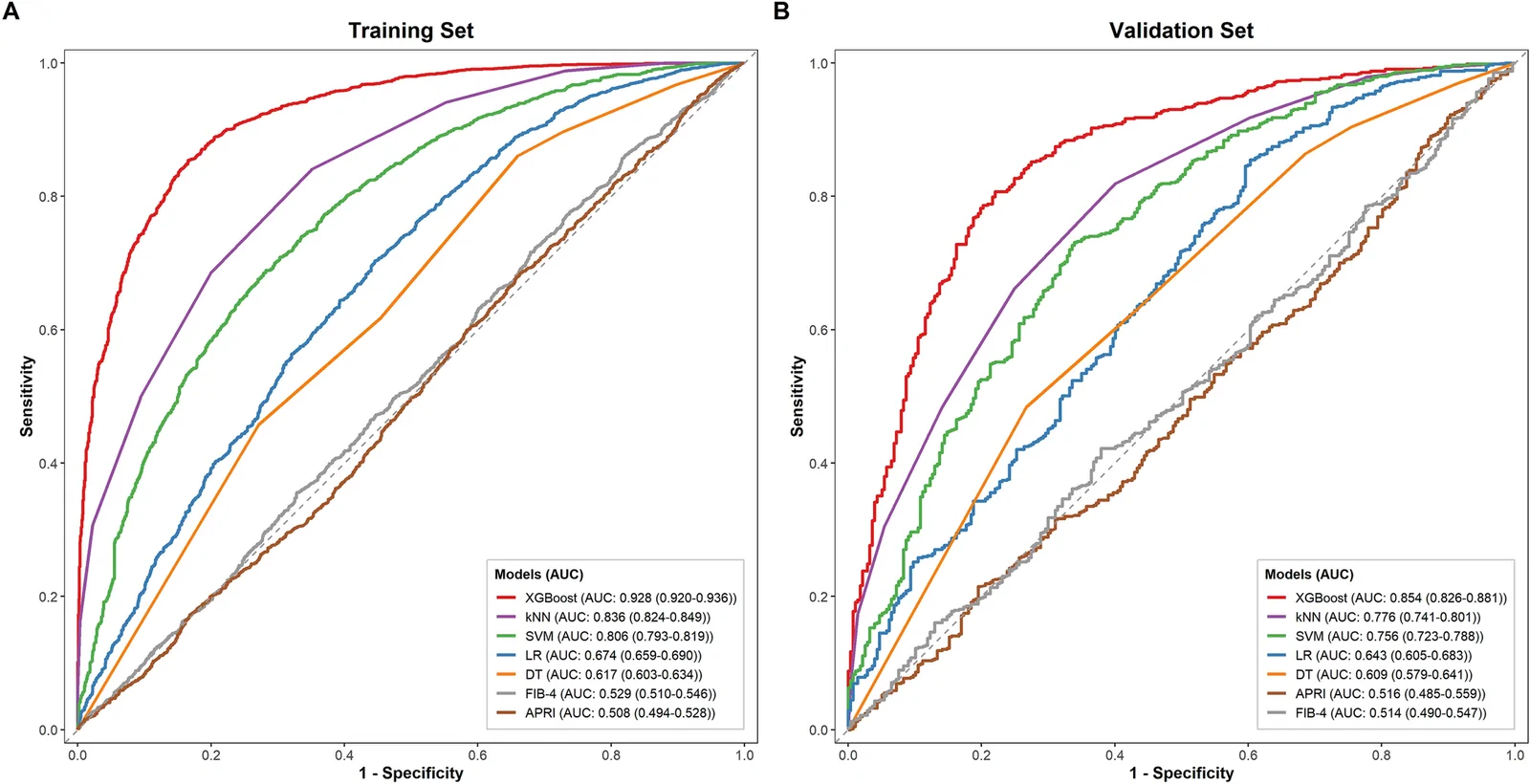
ROC curves of machine learning models and clinical indices. **A** Training set (*n* = 4311); **B** Validation set (*n* = 1847). Red lines represent the XGBoost model, which achieves the highest AUC (0.928 and 0.854, respectively). ROC: receiver operating characteristic; APRI: aspartate aminotransferase to platelet ratio index; FIB-4: fibrosis-4 index; kNN*:* k-nearest neighbor; LR: logistic regression; SVM: support vector machine; DT: decision tree; XGBoost: extreme gradient boosting

Notably, the XGBoost model exhibited a superior diagnostic accuracy compared to conventional non-invasive indices. In the validation set, the XGBoost model exhibited a higher AUC than either FIB-4 (0.514; 95% *CI*: 0.490–0.547; AUC difference = 0.340, Delong’s *Z* = 12.76, *P* < 0.001) or APRI (0.516; 95% *CI*: 0.485–0.559; AUC difference = 0.338, Delong’s *Z* = 12.42, *P* < 0.001), as confirmed by the DeLong test (Fig. 3). These findings suggest that the ML model outperforms traditional scoring systems in predicting schistosomiasis-related liver fibrosis.

**Fig. 4:**
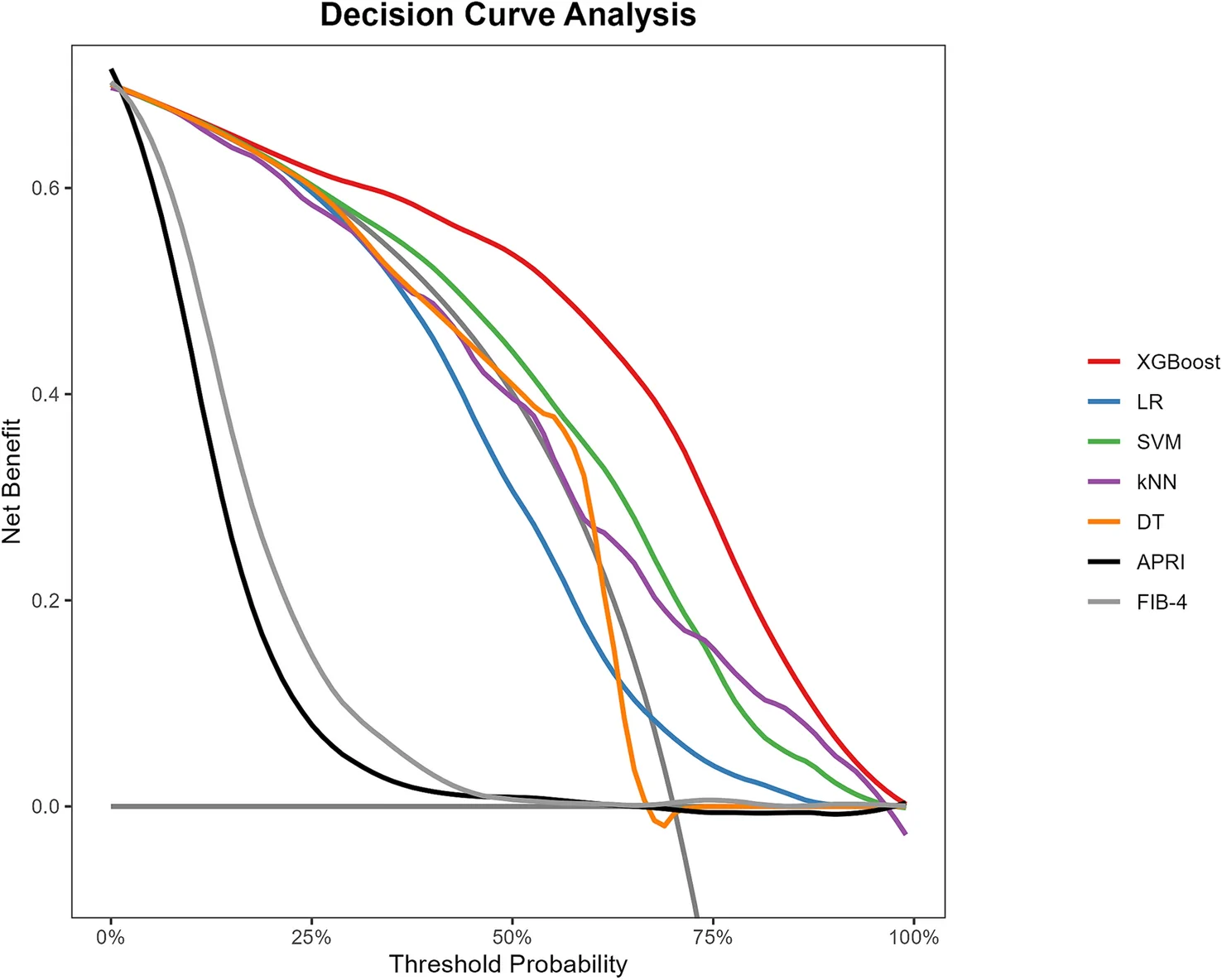
DCA of various machine learning models and clinical indices for predicting liver fibrosis. Horizontal gray line: 'Treat None' strategy (Net Benefit = 0). X-axis intersections indicate the threshold probabilities beyond which the models provide no net benefit. APRI: aspartate aminotransferase to platelet ratio index; FIB-4: fibrosis-4 index; kNN*:* k-nearest neighbor; LR: logistic regression; SVM: support vector machine; DT: decision tree; XGBoost: extreme gradient boosting

To further evaluate the clinical utility of the diagnostic models, DCA was performed to compare the net benefits of five ML models and two clinical indices. As illustrated in Fig. 4, the horizontal gray line represents the 'Treat None' strategy (zero net benefit), and the intersections of the curves with the horizontal axis indicate the threshold probabilities at which the net benefit becomes zero. Notably, the XGBoost model (red line) demonstrated a substantial clinical net benefit across a broad spectrum of threshold probabilities. It consistently outperformed all other comparative models, including SVM, kNN, DT and LR, as well as the traditional APRI and FIB-4 scores. While the traditional clinical indices provided limited net benefit beyond a 25% threshold, the XGBoost model maintained a robust positive net benefit up to a threshold of approximately 95%. These findings indicate that use of the XGBoost model for clinical decision-making would lead to superior outcomes and effectively reduce unnecessary diagnostic procedures.

### Feature contribution analysis using SHAP

**Fig. 5:**
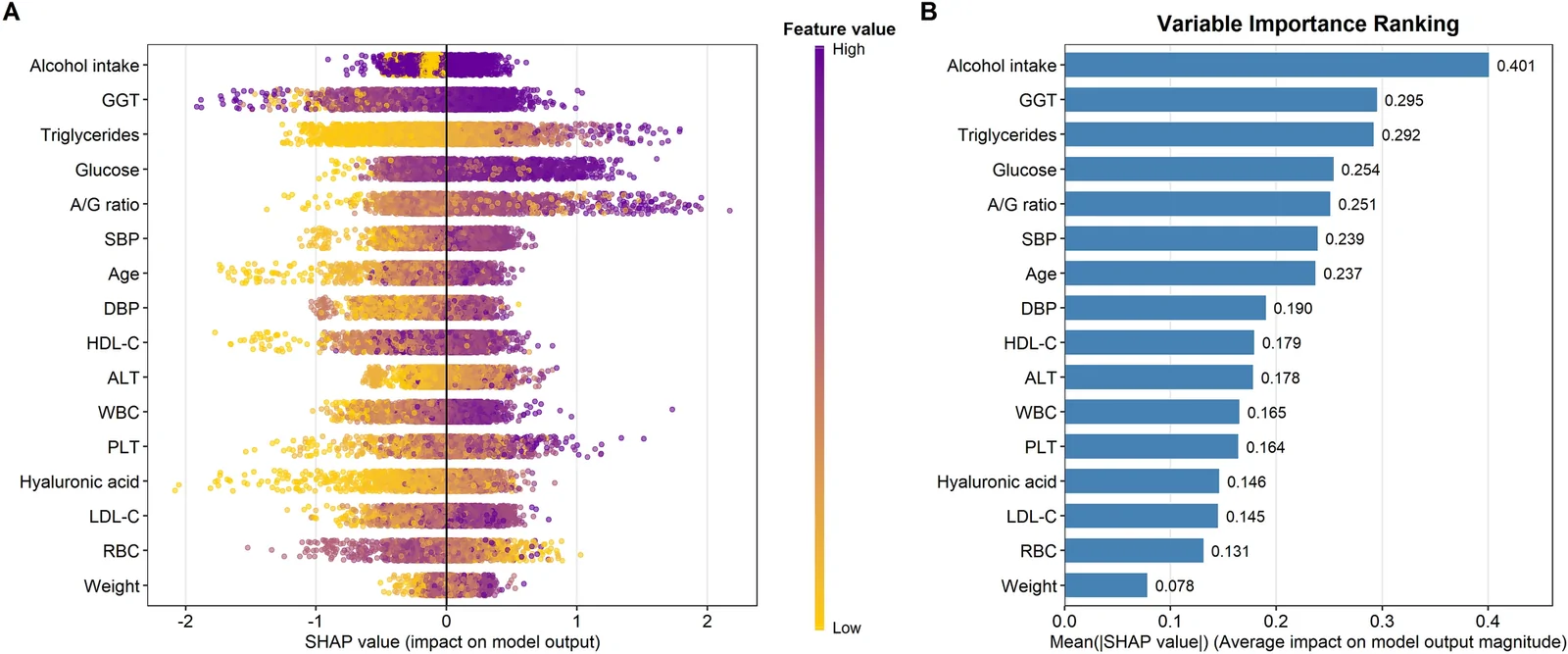
Global interpretation of the XGBoost model via SHAP analysis. **A** SHAP beeswarm summary plot. Variables are ranked by their global importance from top to bottom. Each point represents an individual patient. The horizontal axis depicts the SHAP value, where a positive value (right of the zero line) indicates an increased risk of liver fibrosis, and a negative value (left) indicates a decreased risk. The color gradient on the right of panel A represents the feature value, ranging from low (yellow) to high (purple); B, Variable importance ranking. The bars represent the mean absolute SHAP value for each variable, quantifying the average magnitude of its impact on the model output. SBP: systolic blood pressure; DBP: diastolic blood pressure; RBC: red blood cell; WBC: white blood cell; PLT: platelet; LDL-C: low-density lipoprotein cholesterol; HDL-C: high-density lipoprotein cholesterol; ALT: alanine aminotransferase; GGT: gamma-glutamyl transferase; A/G ratio: albumin/globulin ratio

To elucidate the contribution of each variable to the performance of the XGBoost model, SHAP analysis was conducted. The SHAP summary plot (Fig. 5A) illustrates the individualized impact and directionality of the 16 predictors. Feature values are represented by a color gradient (purple for high, yellow for low), with the horizontal axis indicating the SHAP value. Positive SHAP values denote an increased risk of liver fibrosis. Higher levels of alcohol intake, GGT, and triglyceride were the strongest positive contributors to the predicted risk.

The global variable importance ranking (Fig. 5B), based on mean absolute SHAP values, identified alcohol intake as the most influential predictor (mean SHAP value = 0.401). This was followed by GGT (0.295) and triglyceride (0.292). Additional key contributors included glucose (0.254), the A/G ratio (0.251), SBP (0.239) and age (0.237), while factors such as weight and RBC had the least impact on the final decision-making process of the model.

## Discussion

In this study, we developed and validated five ML models based on epidemiological and laboratory data from 6158 individuals with a history of *S. japonicum* infection. The XGBoost model demonstrated the best performance, achieving an AUC of 0.854 (95% *CI*: 0.826–0.881) in the validation set. This performance was significantly superior to traditional non-invasive models, specifically APRI and FIB-4. Furthermore, DCA confirmed that our models provide a substantial clinical net benefit, underscoring their potential for practical application. These findings establish a robust foundation for developing objective and precise tools to assess liver fibrosis among individuals with *S. japonicum* infection.

Our study highlights the diagnostic limitations of established indices like FIB-4 and APRI in the context of schistosomiasis. While these models are highly accurate for viral hepatitis and non-alcoholic fatty liver disease [41–43], they yielded suboptimal results in our cohort (AUC values of 0.514 and 0.516, respectively). This diagnostic failure is likely rooted in the unique pathophysiological mechanisms of *S. japonicum* infection, which differ fundamentally from viral or alcoholic cirrhosis [44]. Unlike the diffuse hepatocellular damage characteristic of hepatitis, schistosomal egg granulomas concentrate within the portal vein branches, leading to pre-sinusoidal obstruction while relatively preserving hepatocyte function and arterial blood supply [45]. Consequently, biochemical markers heavily weighted in FIB-4 and APRI may not sensitively reflect the specific fibro-vascular remodeling induced by schistosome eggs. To overcome these limitations, we utilized a machine learning approach. The superior performance of XGBoost is largely attributable to its ability to detect complex, non-linear correlations inherent within the clinical dataset. Unlike traditional linear models, XGBoost employs a gradient boosting framework that iteratively minimizes residuals and captures high-dimensional feature interactions, allowing for more accurate modeling of schistosomiasis-related fibrosis compared to traditional linear models [46]. Our findings thus align with the World Health Organization’s emphasis on the necessity of population-specific diagnostic tools to balance accuracy and pragmatism [47].

Advances in ML have increasingly enabled the transformation of vast clinical datasets into actionable diagnostic models [48]. While previous ML models mainly focused on liver fibrosis in viral hepatitis [29, 39, 49] or fatty liver disease [50], these models significantly improve diagnostic accuracy compared to traditional serum indices [38, 40]. However, the diagnostic value of ML in liver fibrosis has not been explored among individuals with prior *S. japonicum* infection. To our knowledge, this is the first ML model specifically developed for predicting liver fibrosis among individuals with a history of schistosomiasis infection. A previous study developed an ML model for active schistosomiasis patients using routine blood markers and achieved an AUC of 0.818; however, that study had a smaller sample size (*n* = 1049) [33]. Our study is much larger (*n* = 6158) and focuses on individuals infected with *S. japonicum* decades ago. Moreover, the progression from infection to fibrosis is a long-term chronic process. By focusing on survivors with prior infection, our study is better positioned to evaluate the performance of the model for early diagnosis of liver fibrosis in a stable, post-treatment population. Although China is approaching the national elimination of schistosomiasis, a substantial population of individuals with prior infections persists [7]. Our XGBoost model relies exclusively on routine epidemiological and laboratory data, offering a more objective and standardized alternative to ultrasonography, which is frequently hindered by high inter-observer subjectivity. This advantage is particularly salient in primary healthcare settings where specialized radiological expertise is often scarce. By utilizing these accessible indicators, the model functions as an effective tool for risk stratification. Clinicians can prioritize high-risk individuals for advanced imaging or liver biopsy, while managing those with low predicted fibrosis through less intensive follow-up care. Ultimately, this approach optimizes resource allocation, reduces the necessity for invasive procedures, and facilitates timely anti-fibrotic interventions by improving the early detection of subtle fibrosis.

Furthermore, DCA demonstrated the clinical utility of our model. While APRI and FIB-4 provided limited benefits, the XGBoost model showed a robust net benefit across a wide range of threshold probabilities (up to 95%). This indicates that our model can effectively support clinical decision-making and reduce unnecessary diagnostic procedures. Model interpretability analysis using SHAP values revealed that alcohol intake, GGT, and triglycerides were the top three predictors. It is important to note that these findings represent statistical associations identified by the machine learning model rather than established causal relationships [51]. However, the high predictive weight of alcohol intake in our model suggests that it is a key feature associated with the fibrotic status in this specific cohort. Therefore, while further longitudinal research is required to confirm causality, our results suggest that monitoring lipid levels and considering lifestyle factors, such as alcohol intake, may provide practical value for the risk assessment and clinical management of schistosomiasis patients. Furthermore, chronic hypertriglyceridemia promotes liver injury through lipid accumulation, oxidative stress, and inflammation in metabolic dysfunction-associated steatotic liver disease [52]. Our results suggest that controlling lipid levels is also vital for improving liver health in this specific population. Additionally, elevated GGT may reflect abnormal glutathione metabolism and oxidative damage, which further accelerates the fibrotic process.

Despite these strengths, our study has several limitations. First, the study population was limited to Jiangsu Province. This geographic restriction may affect the generalizability of the model. Future studies retrieving data from other endemic provinces across China are required to validate our findings. Second, due to the cross-sectional design, some epidemiological data were collected through retrospective surveys, which may introduce recall bias. In addition, data on liver status prior to the initial infection were unavailable. We could not exclude participants who had liver fibrosis before they were infected. However, the average age at initial diagnosis in this study was approximately 17 years, suggesting that the presence of pre-existing liver fibrosis at that stage was highly unlikely. Furthermore, individual records regarding treatment history were unavailable; we could not compare the risk of fibrosis between patients with successful treatment and those with treatment failure. While this represents a data gap, our model is designed to provide a pragmatic assessment of a patient’s current fibrotic status, which remains the immediate priority in clinical screening. Finally, while our XGBoost model demonstrated excellent performance, it is currently a computational algorithm that lacks a user-friendly interface for direct bedside application. Future work should focus on the clinical translation of this algorithm into digital decision-support systems, such as web-based diagnostic calculators. These objective, data-driven platforms would enable primary care physicians to efficiently identify high-risk individuals among those with prior *S. japonicum* infection.

## Conclusions

We developed and validated an XGBoost-based model that outperforms traditional non-invasive indices for identifying liver fibrosis among individuals with prior *S. japonicum* infection. This model represents a promising step toward a digital diagnostic tool that can assist clinicians in grading fibrosis, optimizing early interventions, and monitoring treatment outcomes in schistosomiasis-endemic areas.

## Data Availability

All data presented in this study are available upon request by contact with the corresponding author.

## Abbreviations

A/G ratio: Albumin/globulin ratio
ALD: Alcohol-associated liver diseases
ALT: Alanine aminotransferase
APRI: Aspartate aminotransferase to platelet ratio index
AST: Aspartate aminotransferase
AUC: Area under the receiver operating characteristic curve
BMI: Body mass index
*CI*: Confidence interval
DBP: Diastolic blood pressure
DCA: Decision curve analysis
*df*: Degrees of freedom
DT: Decision tree
FIB-4: Fibrosis-4 index
GGT: Gamma-glutamyl transferase
GVIF: Generalized variance inflation factor
HDL-C: High-density lipoprotein cholesterol
IQR: Interquartile range
kNN: K-nearest neighbor
LDL-C: Low-density lipoprotein cholesterol
LR: Logistic regression
MASLD: Metabolic dysfunction-associated steatotic liver disease
ML: Machine learning
NPV: Negative predictive value
*OR*: Odds ratio
PLT: Platelet
PPV: Positive predictive value
RBC: Red blood cell
ROC: Receiver operating characteristic
SBP: Systolic blood pressure
*SD*: Standard deviation
*SE*: Standard error
SHAP: Shapley additive explanations
SVM: Support vector machine
VIF: Variance inflation factor
WBC: White blood cell
XGBoost: Extreme gradient boosting
